# Catalytic Reduction of Aromatic Nitro Compounds to Phenylhydroxylamine and Its Derivatives

**DOI:** 10.3390/molecules29184353

**Published:** 2024-09-13

**Authors:** Min Yu, Dachen Ouyang, Liqiang Wang, You-Nian Liu

**Affiliations:** 1 Hunan Provincial Key Laboratory of Micro & Nano Materials Interface Science, College of Chemistry and Chemical Engineering, Central South University, Changsha 410083, China; 222301043@csu.edu.cn (M.Y.); ouyangdachen@163.com (D.O.); 2 Henan Province Industrial Technology Research Institute of Resources and Materials, School of Material Science and Engineering, Zhengzhou University, Zhengzhou 450001, China

**Keywords:** phenylhydroxylamine, aromatic nitro compounds, catalytic reduction, reduction mechanism

## Abstract

Phenylhydroxylamine and its derivates (PHAs) are important chemical intermediates. Phenylhydroxylamines are mainly produced via the catalytic reduction of aromatic nitro compounds. However, this catalytic reduction method prefers to generate thermodynamically stable aromatic amine. Thus, designing suitable catalytic systems, especially catalysts to selectively convert aromatic nitro compounds to PHAs, has received increasing attention but remains challenging. In this review, we initially provide a brief overview of the various strategies employed for the synthesis of PHAs, focusing on reducing aromatic nitro compounds. Subsequently, an in-depth analysis is presented on the catalytic reduction process, encompassing discussions on catalysts, reductants, hydrogen sources, and a comprehensive assessment of the merits and drawbacks of various catalytic systems. Furthermore, a concise overview is provided regarding the progress made in comprehending the mechanisms involved in this process of catalytic reduction of aromatic nitro compounds. Finally, the main challenges and prospects in PHAs’ production via catalytic reduction are outlined.

## 1. Introduction

**Scheme 1:**
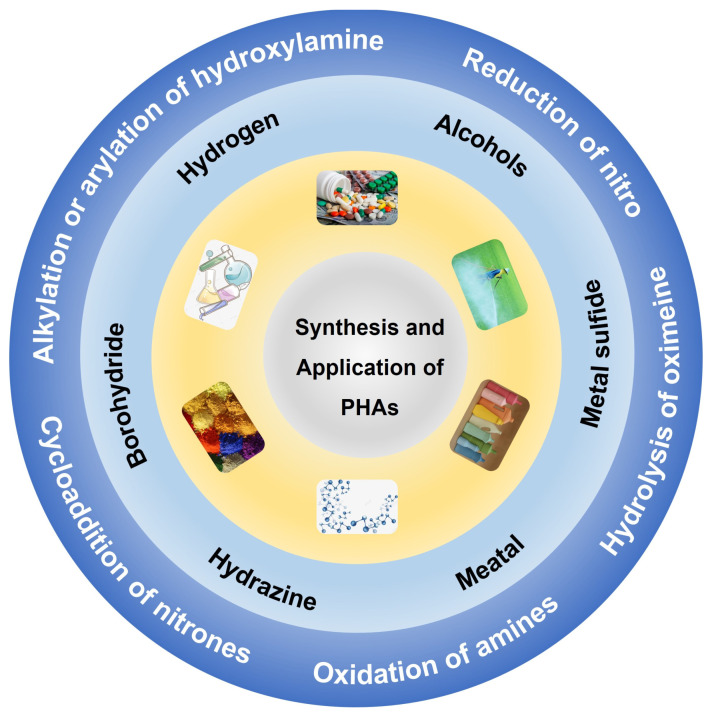
Synthesis methods and applications of PHAs.

Hydroxylamines represent a class of compounds that feature the functional group X-H(OH), where the hydroxyl group (-OH) is attached to an amine group (-NH_2_) [1]. They can exist in both organic and inorganic forms. Hydroxylamines are found in applications in various fields, including chemistry [2], biology [3,4], and materials science [5,6]. N-phenylhydroxylamine (N-PHA) and its derivates (PHAs), an important member of the hydroxylamine family, have recently received increasing attention. In 1894, N-PHA was serendipitously discovered by Wohp and Barnbergers independently while engaging in the reduction of nitrobenzene using zinc powder [7]. This finding resulted in the identification of a notable rearrangement process known as the Bamberger rearrangement, which involves the conversion of N-PHA into 4-aminophenol under given conditions within a strongly acidic aqueous medium. This reaction provides important theoretical support for introducing amino or other nucleophilic reagents on aromatic compounds. Now, N-PHA and PHAs have found wide applications in chemistry [8], biology [9], medicine [10], and pharmaceuticals [11] (Scheme 1). For example, N-PHA and PHAs are important intermediates for the synthesis of various drugs [12] and pesticides [13], bioactive molecules, organic synthesis reagents, polymerization inhibitors [14], electronic industrial supplies, cosmetics, etc. Notably, N-PHA is widely explored for the synthesis of acetaminophen [15], also known as paracetamol; its production in 2020 reached 176.8 kt.

Diverse methods have been developed for the synthesis of PHAs, including hydrolysis of oximes [16], oxidation of amines [17], substitution [18], addition reaction [19], and reduction methods (Scheme 1). Of all the methods, reducing aromatic nitro compounds (e.g., nitrobenzene) represents the most efficient approach and is the most exploited in industrial production. Depending on whether a catalyst is utilized, the reduction process can be defined as chemical reduction or catalytic reduction. Also, the reduction process varies according to the reductants or H resources utilized. Catalytic reduction involves catalytic hydrogenation using H_2_ as both the reductant and the H donor and catalytic hydrogen transfer using chemicals containing active hydrogen species (e.g., methanol). Also, electrocatalysis, photocatalysis, and biocatalysis were reported to reduce aromatic nitro compounds by affording “electrons” as reductants and H-containing compounds (e.g., H_2_O) as H donors. The nitro reduction generally undergoes a perplexed process, and PHAs are usually an intermediate and can be further reduced to anilines; coupling reactions can also happen between different derivates to generate bimolecular products [20,21]. As a result, controlling the reduction process to afford N-PHA and its derivatives selectively is crucial but still challenging.

The “control procedure” for non-catalytic reduction depends on strictly maintaining the stoichiometric ratio of reductants to nitro compounds. Also, suitable reaction systems, including reductants, temperature, solvents, and reaction time, should be selected to differentiate the energy barrier of the reaction between producing N-PHA and by-products, hence selectively affording PHAs products. Unfortunately, the non-catalytic reduction process needs to consume many reductants and inevitably generate numerous by-products and waste, posing significant challenges in product purification and leading to various environmental problems. The catalytic reduction was first reported in the early 20th century, when it was discovered that noble metals (such as rhodium, iridium, palladium, platinum, etc.) or metal complexes could catalyze the hydrogenation of nitrobenzene to produce PHAs under hydrogen [22]. Later on, it was discovered that non-noble metals and certain non-metallic matrices could also catalyze the hydrogenation process and the hydrogen resources, including H_2_, borohydride, hydrazine hydrate, and alcohol.

Despite the extensive efforts in developing catalysts for reducing aromatic nitro compounds to produce N-PHA and its derivatives, there remains significant potential for improvement in its catalytic performance. For example, Raney nickel is widely utilized for the synthesis of PHAs due to its relatively low cost. However, to improve selectivity, hydrazine hydrate was commonly used as a reducing agent and reacted at low temperatures, inevitably increasing the preparation cost and environmental burden. Moreover, the production process is still carried out in non-continuous kettle reactors. In addition, the mechanism of the catalytic reduction of nitroaromatics (NA) to N-PHA is still unclear, posing a great challenge for designing catalysts. Clearly, a review focused on the development process of synthesizing N-PHA or PHAs via catalytic reduction of nitro compounds holds significance in terms of its environmentally friendly and cost-effective manufacturing. Herein, a review of the progress in the synthesis of N-PHA and its derivatives through the catalytic reduction of nitro compounds is presented. Initially, it outlines the commonly employed synthetic techniques for hydroxylamine, while emphasizing nitro compound reduction. The discussion delves into the catalytic reduction methods, focusing on the impact of catalysts, reductants, hydrogen sources, and reaction conditions (such as solvents and temperature) on the reduction process. Furthermore, various catalytic reduction mechanisms are summarized. Ultimately, the main challenges and prospects faced in the synthesis of PHAs are outlined.

## 2. Synthesis Strategy for Hydroxylamine

### 2.1. Alkylation or Arylation of Hydroxylamine

**Scheme 2:**
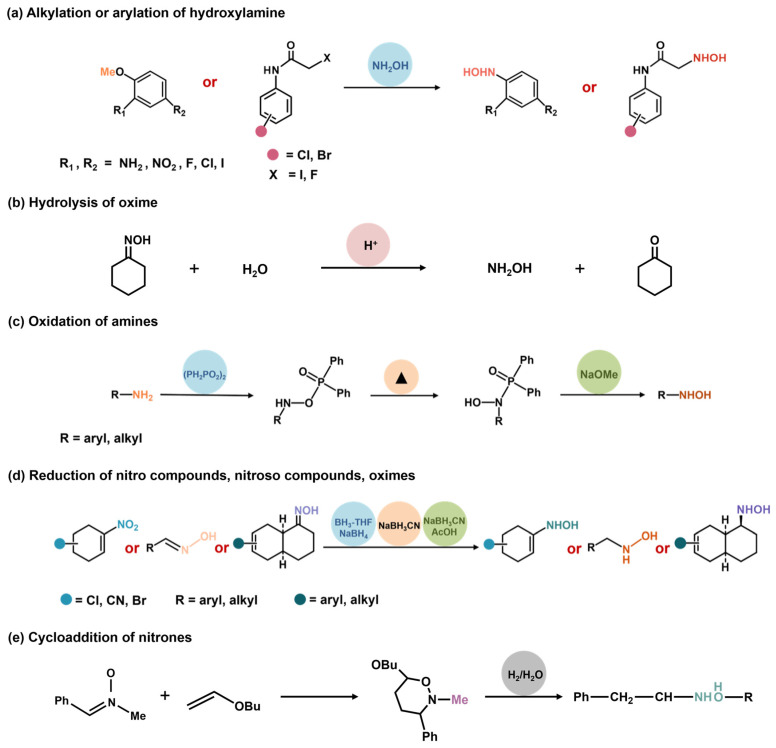
Synthesis strategies for hydroxylamine.

Alkylation or arylation of hydroxylamine is an early method utilized to obtain complicated hydroxylamine compounds (Scheme 2a). This process involves the reaction between alkyl or aryl compounds with hydroxylamine [23,24]. The nucleophilic reagent hydroxylamine, containing N-O bonds, can react with electrophilic reagents (alkyl compounds/aromatic compounds) to produce corresponding hydroxylamine compounds. In this method, the yield and selectivity of hydroxylamine and its derivatives are severely affected by the reaction conditions. Therefore, it is necessary to control the reaction conditions strictly. For example, the solvent should have good solubility in hydroxylamine and alkyl or aryl compounds. Usually, a high temperature, high pressure, and an extended reaction time are required in these reactions [25]. In addition, the reaction involves tedious separation and purification processes, resulting in high costs and energy consumption.

### 2.2. Hydrolysis of Hydroxamic Acid, Oxime, or Nitrone

Hydrolysis substrates, such as hydroxamic acid, oxime, or nitrones, can lose a hydroxyl group under the attack of water molecules under suitable pH conditions, affording hydroxylamine or hydroxylamine compounds [26,27]. The hydrolysis reaction can take place under mild conditions, without the need for harmful or expensive catalysts. It does not produce other by-products [28]. However, hydrolysis has its limitations and problems. The reaction efficiency is not very high, as it is strongly affected by thermodynamic equilibrium [16]. During the reaction process, some hydroxylamine can be destroyed or transformed, affecting the final product’s purity and yield. Additionally, subsequent separation and purification steps are required, thus increasing the overall cost and energy consumption of the processes.

### 2.3. Oxidation of Amines

Amino oxidation is a promising method for the synthesis of hydroxylamines (Scheme 2b). Various primary and secondary amines can be oxidized into their corresponding hydroxylamines using different oxidants [12]. For instance, the primary amines can be selectively converted to hydroxylamines in a sodium tungstate complex with hydrogen peroxide/urea (UHP) [29]. However, this method depends on the availability of substrates, and the primary hydroxylamines can undergo further oxidation to nitroso and nitro compounds, resulting in low yield and selectivity [30]. Due to the limitations of the substrate, this method cannot be applied in industrial production.

### 2.4. Reduction of Nitro Compounds, Nitroso Compounds, Oximes, and Oxime Ethers

There are few reports on the reduction of nitroso compounds to hydroxylamine, as nitroso compounds are more difficult to obtain than their corresponding hydroxylamine counterparts [31]. Aromatic nitroso compounds can be reduced to hydroxylamine in the presence of ascorbic acid, glyoxylate, and nicotinamide adenine dinucleotide (NADH). Oxime, oxime ether, and nitroketone can be reduced to hydroxylamine by using complex hydrides such as sodium cyanide borohydride as reducing agents (Scheme 2c) [32]. In addition, nickel/iridium catalysts can catalyze asymmetric hydrogenation of oxime to hydroxylamine [33]. This reaction has a narrow substrate scope and requires harsh conditions, such as high-pressure hydrogen or strong reducing agents. During the process, some side reactions may occur (e.g., N-O bond cleavage and C-N bond hydrogenolysis), resulting in the low yield and purity of hydroxylamines.

### 2.5. Cycloaddition of Nitrones, Oximes, and Nitroso Compounds

The cycloadditions (e.g., [4 + 2] and [3 + 2]) are mainly utilized for the synthesis of cyclic hydroxylamines [34]. For instance, benzene can react with hydroxylamine via bimolecular cycloaddition to produce N-PHA. From the viewpoint of molecular orbital theory, in such a reaction, the highest occupied orbital (HOMO) of benzene interacts with the lowest unoccupied orbital (LUMO) of hydroxylamine, forming two new σ bonds and a six-membered ring. This method streamlines the reaction steps and minimizes the formation of by-products and waste, thus adhering to the principles of green chemistry [35]. However, it necessitates special catalysts and reaction conditions, demands high activation of benzene and stability of hydroxylamine, and suffers low selectivity and yield. Table 1 summarizes the differences in the main research methods for synthesizing hydroxylamine. Through these comparisons, the advantages and disadvantages of various methods can be presented more intuitively.

## 3. Survey of Reducing Agents and Hydrogen Sources

### 3.1. Survey of Reducing Agents

Nitro compound reduction is the most used method for the synthesis of N-PHA and PHAs, and common reducing agents include metals, hydrazine, borohydride, hydrogen, etc. [36].

#### 3.1.1. Metal Reducing Agents

Metals such as Zn, Sn, Fe, and Sm have been widely utilized to reduce nitro compounds. The metal donates electrons to nitro compounds in the presence of an H donor (e.g., H_2_O), thereby reducing them to hydroxylamine groups. The selection of metal reducing agents is related to their reducing ability, while the reducing ability of metals is directly related to the standard electrode potential. In addition, reaction conditions, such as the solvent, temperature, and reaction time, can affect the reduction process. Therefore, the reaction process can be controlled by selecting appropriate metals and optimizing reaction conditions. However, metal reduction reactions are prone to producing a series of by-products, such as metal oxides and metal salts. These by-products pose a significant challenge for separating and purifying PHAs.

#### 3.1.2. Hydrazine Reducing Agent

**Scheme 3:**
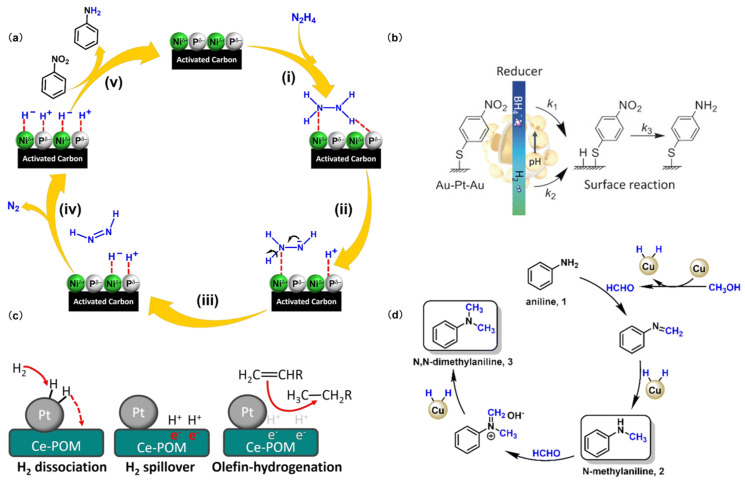
Mechanism of action of different reducing agents. (**a**) (i) The combination of catalyst and N_2_H_4_, (ii–iv) nitrogen and hydrogen in N_2_H_4_ undergo heterolysis, (v) hydrogenation of Nitrobenzene. Reproduced with permission from Ref. [38]. Copyright 2024 Elsevier. (**b**) The reaction mechanism of sodium borohydride driven platinum catalyzed reduction of nitro compounds. Reproduced with permission from Ref. [40]. Copyright 2016 Wiley-VCH. (**c**) Hydrogen overflow reduction mechanism on the surface of modified Pt nanoparticles. Reproduced with permission from Ref. [42]. Copyright 2023 Wiley-VCH. (**d**) The reaction pathway for the N-methylation of amines with methanol over CuCo catalysts. Reproduced with permission from Ref. [44]. Copyright 2022 The Royal Society of Chemistry.

Hydrazine exhibits notable reducing capabilities due to its N-H bonds, which can undergo cleavage to liberate hydrogen atoms or ions in suitable conditions. Consequently, this process facilitates the transfer of electrons to the substrates, leading to their reduction. However, pure hydrazine is toxic and hazardous, and its hydrated variant, alongside derivatives such as methyl hydrazine and hydrazine sulfate, is often employed as a relatively safer alternative. Particularly, hydrated hydrazine (N_2_H_4_·H_2_O) is a prevalent liquid reducing agent used in both experimental and industrial settings. In an aqueous solution, hydrazine serves as both a reducing agent and a hydrogen carrier [37]. It gradually breaks down into nitrogen and hydrogen atoms (Scheme 3a), facilitating the transfer of hydrogen atoms to nitrobenzene [38]. This process leads to a stepwise reduction of nitrobenzene, producing nitrosobenzene and N-PHA until aniline. Hydrazine as a reducing agent offers several advantages: the reaction process is straightforward; can be conducted at low temperatures; and does not affect other functional groups on the benzene ring, such as cyanide or ester groups. Moreover, the system using hydrazine as a reducing agent is further underscored by its high conversion rates and its clean and safe handling attributes. Nevertheless, the application of hydrazine is accompanied by certain limitations. The inherent toxicity of hydrazine raises safety concerns, and the amines formed as by-products after the reaction are also hazardous, requiring meticulous separation and disposal procedures. Additionally, the utilization of hydrazine results in the production of a substantial amount of wastewater and waste gases, presenting environmental obstacles that need to be addressed.

#### 3.1.3. Borohydride Reducing Agent

Borohydrides such as sodium borohydride (NaBH_4_), potassium borohydride (KBH_4_), and ammonia borane (NH_3_BH_3_) are commonly employed as reducing agents in synthesis. Despite their inherent mildness in reducing capacity, borohydrides can be significantly boosted in reactivity by combining them with transition metals or their salts, resulting in a more potent reducing system [39]. This combination strategy enables the reduction of aromatic nitro compounds to PHAs under optimized conditions. Within the borohydride system, borohydrides can decompose and generate the H atom in water or ethanol (Scheme 3b), which initiates the reduction of nitro compounds [40]. The advantages of utilizing borohydrides include that the reaction goes smoothly under mild conditions, displays rapid kinetics, provides a high product yield, and demonstrates exceptional selectivity. These characteristics render borohydrides highly appealing for applications in synthetic chemistry. However, the relatively high cost of these reagents discourages their use in large-scale or cost-sensitive processes. Additionally, the by-products of the reaction, particularly boric acid salts, can pose an additional challenge in processing, necessitating their removal and disposal.

#### 3.1.4. Hydrogen Reducing Agent

Hydrogen, as a reducing agent in hydrogenation reactions, is a fundamental aspect of various industrial chemical processes, particularly in the large-scale reduction of bulk chemicals. Due to the abundance of H_2_ and the ideal by-product of H_2_O, hydrogenation using H_2_ as a hydrogen donor is more in line with green chemistry and economy. Recently, there has been increasing research interest in using H_2_ to reduce nitro compounds for the preparation of PHAs. However, H_2_ is often chemically inert, and catalysts are required to facilitate its reducing process. In the presence of a catalyst, H_2_ undergoes either homolysis or heterolysis on the active species [41,42], generating highly reactive hydrogen atoms that react with nitro compounds (Scheme 3c), leading to the gradual conversion of nitro compounds into hydroxylamine groups. Therefore, H_2_ as a reducing agent, is mainly utilized in catalytic reduction instead of chemical reduction. This approach is known for its high efficiency, environmental friendliness, and cost-effectiveness.

#### 3.1.5. Other Reducing Agents

Metal sulfides such as sodium sulfide (Na_2_S), ammonium sulfide ((NH_4_)_2_S), and ammonium hydrogen sulfide (NH_4_HS) could also be utilized as reducing agents and has a long-standing history in chemical synthesis [43]. As early as 1921, Haworth and coworkers reported using these reagents to obtain hydroxylamine, achieving yields ranging from 15% to 62%. Metal sulfide can dissociate into S^2−^ in solution and further hydrolyze to obtain HS^−^ ions. Based on the differences in surface properties of catalysts and the content of nitro compounds, different reactive reducing sulfur species (S^2−^, HS^−^, S2^2−^, S3^2−^ and S^0^) are induced. The reactive reducing sulfur species could reduce nitro chemicals to hydroxylamine, aniline, or/and other products.

The proton solvent provides H atoms, and the sulfide ions produced act as reducing species to reduce nitro compounds, gradually hydrogenating to form nitroso and hydroxylamine compounds. This method boasts simplicity, mild reaction conditions, and cost-effectiveness. However, it also suffers from a low yield of the desired product and generates substantial by-products, like sulfates.

Alcohols such as methanol [44], ethanol, and isopropanol are also common reducing agents in catalytic hydrogenation processes (Scheme 3d) [45]. Despite their potential, systematic studies on the use of alcohol-based reducing agents for the synthesis of PHAs, are limited. More comprehensive research is needed to optimize this method, focusing on enhancing reaction rates, minimizing the side reactions, and improving PHA yields and product purity.

### 3.2. Survey of Hydrogen Source

Hydrogen sources could come from either solvents or reducing agents. For the non-catalytic reduction process, H mainly comes from solvents utilized, such as water, alcohol, etc. For catalytic hydrogenation, reducing agents usually serve as hydrogen sources. In the direct hydrogenation process, H_2_ is the H donor; meanwhile, during the catalytic transfer hydrogenation process, borohydride, hydrazine hydrate, and alcohols work as hydrogen sources [46].

## 4. Non-Catalytic Synthesis of PHAs

### 4.1. Metal Reducing Agent

**Figure 1:**
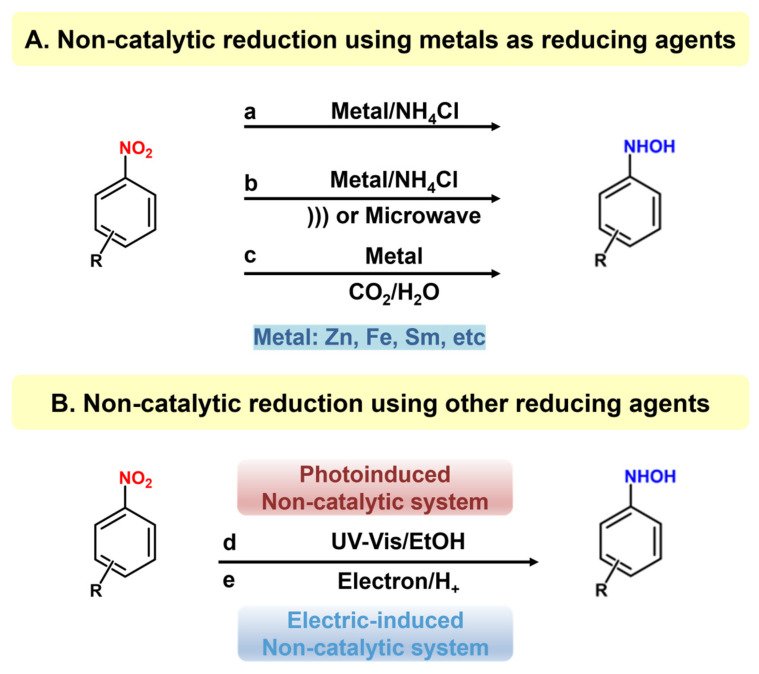
Non-catalytic reduction methods for preparing PHAs.

Metal reduction commonly involves the reduction of nitro compounds using low-valence metals in an electrolyte-containing aqueous solution, where water acts as both the solvent and the hydrogen source. The reduction process occurs at the interface, with the metal serving as the electron donor and the nitro compound accepting electrons and protons to form hydroxylamine. This exothermic reaction can elevate the reaction temperature, potentially causing the over-reduction of hydroxylamine to form amines or oxidation to form by-products like azobenzene derivatives. To prevent these outcomes and ensure high purity and yield of PHAs, precise control of the feed rate between substrates and reductants is essential. Historically, Bambergers first proposed using zinc powder and ammonium chloride for the synthesis of N-PHA via reducing nitrobenzene (Figure 1A), but with a limited selectivity of N-PHA (57%). Similarly, Kamm observed that reaction temperature significantly influences N-PHA yields, with optimal yields ranging from 60% to 70% achieved at temperatures between 20 and 60 °C. This method continues to be a subject of extensive investigation in laboratory settings. For example, Wohl enhanced the yield of N-PHA to 75% by introducing calcium chloride as a promoter into an aqueous solution of ethanol, while Goldschmidt successfully achieved selective PHAs reduction without generating by-products by utilizing zinc powder in a mixture of water and ether with calcium chloride.

Moreover, ultrasound or microwave radiation has been explored to improve the selectivity and activity of the metal-participated reduction process for the synthesis of PHAs (Figure 1A(b)). This approach leverages the radiation and thermal impacts of ultrasound/microwave to stimulate and distribute the metal-reducing agents, augment the interaction between metal and nitro compounds, and accelerate the reaction rate. Additionally, this technique can mitigate the undesired side reactions like over-reduction or coupling, thereby enhancing the selectivity. Ung et al. reported ultrasound-assisted nitroaromatics reduction to prepare the corresponding PHAs [47]. Using 4-hydroxyamino-benzoic acid–ethyl ester as a model substrate, the reaction efficiency was significantly enhanced when activated by ultrasound. This approach demonstrated a rapid reaction rate and high selectivity, efficiency, and adaptability for a range of nitroaromatic compounds containing electron-withdrawing substituents, resulting in favorable yields. However, nitroaromatics with electron-donating groups can be over-hydrogenated to form the corresponding aniline products. The main problem is that Zn powder must be added slowly in batches to control the reaction process, making the operation cumbersome. Shi et al. addressed this issue by employing an ultrasound-assisted technique to reduce nitroaromatics for the synthesis of N-PHA using zinc and formamide under ambient conditions [48,49]. The reaction achieved a N-PHA yield of 91% within a 40-min timeframe. Furthermore, this approach exhibited notable chemical specificity and accommodated nitro compounds containing various subtle functional groups, resulting in a yield of over 90% for the corresponding PHAs.

Microwave is an electromagnetic wave with a frequency of 0.3–300 GHz and a wavelength of 0.001–1 m. Microwave action can cause a higher temperature and pressure in liquids, which can change the kinetic and thermodynamic parameters in the reaction process and thus alter the progress and direction of the reaction. Keenan et al. studied the reduction of nitroaromatics to the corresponding PHAs using Fe or Zn under mild microwave heating conditions [50]. The reaction was microwaved at 120 °C for 5 min, and the PHA yield reached 91%. This method has high PHA yields (80–99%) for nitro compounds containing different functional groups, demonstrating good substrate applicability. In a metal-participated reduction reaction, NH_4_Cl is commonly added to provide an acidic environment to promote the dissolution of the metal and facilitate electron transfer, thereby improving the performance of the reaction. However, introducing NH_4_Cl can increase the cost and difficulty of waste disposal. Considering the above problems, Liu et al. proposed using a series of CO_2_/H_2_O systems as an environmentally friendly alternative (Figure 1A(c)), exhibiting that CO_2_ played an essential role in the selective reduction of nitrobenzene (NB) to N-PHA, avoiding the disadvantages associated with NH_4_Cl [51]. Without CO_2_, only a negligible reduction took place, while under 0.1 MPa CO_2_ (Zn/NB = 3, room temperature), NB was efficiently reduced to N-PHA with 88% yield. The reaction could proceed under normal CO_2_ pressure but with a lower yield. This system was applied to nitroarenes containing other reducible functional groups, giving the corresponding PHAs 88–95% selectivity. Another study investigated the effect of ethanol on hydrogenation and found that when an appropriate amount of ethanol was added, the selectivity of PHAs was significantly promoted [52]. In addition, the relevant work showed that, with the assistance of ultrasound, the required reaction time is shortened, the consumption of Zn is reduced [53], and the yield of PHAs increases. This method offered an environmentally friendly way for the synthesis of PHAs.

Although the metal reduction method offers numerous advantages, including simplicity, mild conditions, and good selectivity, significant obstacles exist concerning reaction control and environmental impact, and better reduction systems are still highly required. The method is highly related to the experimental conditions, such as the pH of the solution, reaction temperature, and reaction time, necessitating strict operational protocols. For example, low-pH solutions often afford aromatic amines, while high-pH solutions tend to result in nitrosobenzene. As for the temperature, a high temperature leads to over-hydrogenation, whereas a low temperature leads to low activity. Moreover, the nature of substituents on the aromatic ring influences the reaction outcome, generally favoring electron-withdrawing groups. In light of these factors, non-catalytic metal reduction is rarely used in industry now.

### 4.2. Other Reducing Agents

Besides metals, reducing agents like borohydride have been previously explored to reduce nitro compounds and produce PHAs. Mourad et al. utilized NaBH_4_ and borane to reduce a β-unsaturated nitro compound. Through the influence of borohydride, borane offers reducibility and directly reduces nitro compounds, resulting in a process that is easily separable and produces high yields of PHAs [54]. However, this approach is limited by its selectivity towards specific substrates, thereby restricting its widespread applicability.

**Figure 2:**
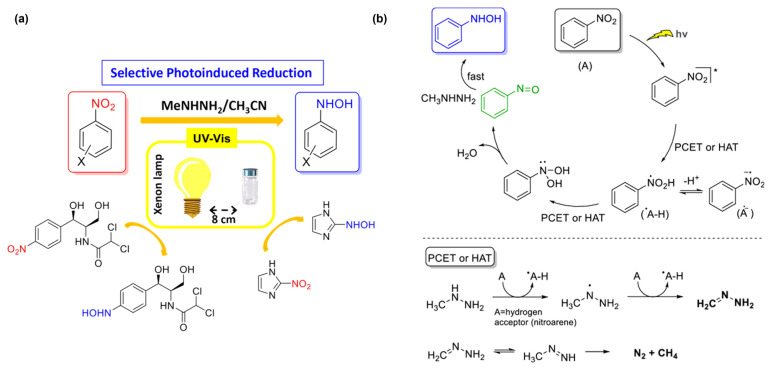
(**a**) Schematic diagram of selective photo induced reduction of nitroaromatic hydrocarbons to N-PHA. (**b**) The Mechanism for the photoinduced synthesis of N-PHA from Nitrobenzene. Reproduced with permission from Ref. [57]. Copyright 2020 American Chemical Society.

Unlike metal reducing agents, non-metal reductants usually possess weak reducing properties. Thus, the extra driving force, like light or electricity, is often exerted to help enhance the reduction ability (Figure 1B(d,e)) [55]. Although alcohols or hydrazines possess limited reducing properties, upon light excitation, they can generate active H atoms, which act as stronger reducing agents and reduce nitro compounds to PHAs. Kaneko and coworkers made a pioneering attempt and investigated the photochemical reduction of 4-nitropyridine-1-oxide to the corresponding PHAs in ethanol [56]. Ethanol can generate active hydrogen when exposed to light, facilitating the hydrogenation of substrates to produce the corresponding PHAs. Furthermore, hydroxylamine undergoes rapid oxidation in the presence of oxygen, prompting further investigation into the resulting oxidation products. Kallitsakis et al. explore methyl hydrazine as a potential reductant [57]. Upon light irradiation, proton-coupled electron transfer (PCET) or hydrogen atom transfer (HAT) occurs between nitroaromatic hydrocarbons and methylhydrazine, generating nitrosoarenes in situ and subsequently producing PHAs, with a yield of up to 99%. The reaction demonstrated a notable selectivity towards PHAs (Figure 2a), with minimal generation of by-products and the preservation of other reducible functional groups on the benzene ring. The influence of solvents has been assessed, noting that while the reaction could proceed in different solvents, its selectivity was significantly reduced in ethyl acetate, and no reaction occurred in pure water. Based on experimental data, the mechanism and pathway of reduction were proposed (Figure 2b).

The electricity-driven reduction reaction was also reported for the synthesis of PHAs from nitro compounds. During the electrochemical reduction process, active atomic hydrogen was first generated in situ on the cathode using water and other sacrificial agents in the electrolyte as hydrogen sources, and electrons supplied by the electrode act as reducing agents [58]. The active H species could reduce aromatic nitro compounds via the proton–electron concerted transfer (CPET) pathway on the cathode surface, resulting in PHAs. The chemical and electrochemical reduction of nitrobenzene would undergo a similar procedure [59,60]. The pioneering endeavors were launched by Bambergers, who exploited electrochemical reduction to synthesize N-PHA at low temperatures using a zinc sheet as the electrode and an ammonium chloride aqueous solution as the cathode solution. Though the N-PHA yield was <29%, such a method provided a basis for the subsequent electrochemical reduction methods. Subsequently, Abdollahi found that numerous factors, including the reactor design, electrolyte composition, agitation rate, and electrochemical parameters like voltage and current density, could influence the yield [61]. Careful optimization of these variables can enhance N-PHA production. Cyr et al. investigated the electrochemical reduction of various related compounds using copper and nickel electrodes in neutral and alkaline methanol aqueous solutions [60]. The voltammetric studies revealed that nitrobenzene, azobenzene, and azoxybenzene underwent electron transfer before the medium’s discharge potential, but N-PHA and hydrazobenzene did not. Aniline could adsorb on the electrode surface, hindering the further hydrogenation of N-PHA, while this effect diminished at lower potentials. To explore the mechanism of electro-reduction of nitrobenzene to prepare PHAs, Seshadri reported the electrochemical reduction of nitrobenzene on a Mo electrode in an aqueous solution and demonstrated that nitrobenzene was reduced on a molybdenum electrode over a wide pH range (pH 4.4–13) [62]. Meanwhile, under alkaline conditions, the reduction stopped at the four-electron stage, and N-PHA did not undergo further reduction.

Non-catalytic reduction holds significant practical value for the synthesis of PHAs due to its operational simplicity, cost-effectiveness, and mild reaction conditions. However, this approach is hampered by the reaction rate and demands precise control over conditions to maintain selectivity, often resulting in over-hydrogenation and unwanted side reactions. Though by integrating technologies such as microwave or ultrasound, the non-catalytic reduction process can be improved to some extent, it is still a great challenge for synthetic PHAs to meet the requirements for industrial production, especially in atom economy and green chemistry.

## 5. Catalytic Synthesis of PHAs

Catalytic reduction is currently the widely exploited method for the preparation of PHAs from aromatic nitro compounds in industry. Noteworthy for its ease of operation and gentle reaction conditions, the catalytic reduction is versatile, accommodating a variety of substituents on the benzene ring, such as unsaturated bonds and halogens, as well as other groups sensitive to reduction. However, the hydrogenation of nitro groups typically occurs as a series of continuous reactions, and avoiding excessive hydrogenation remains challenging. Achieving a high yield of hydroxylamine through selective hydrogenation is rare and remains a significant obstacle in the field. This difficulty underscores the need for improved catalyst design and reaction conditions to enhance selectivity and prevent the formation of over-hydrogenation. The catalyst can be divided into noble-metal, non-noble-metal, and non-metal catalysts. Reductants and H resources utilized in non-catalytic reduction can also be applied to catalytic reduction. Note that hydrogen can serve as both a reductant and a source of hydrogen atoms in catalytic reduction processes.

### 5.1. Noble-Metal Catalyst

#### 5.1.1. H_2_ Reducing Agent

Early research focused on noble-metal catalysts such as platinum (Pt), palladium (Pd), and ruthenium (Ru), which exhibited significant catalytic activity. Consequently, hydrogenation reduction catalyzed by noble metals can occur under gentle conditions, thereby benefiting improving catalytic selectivity.

**Figure 3:**
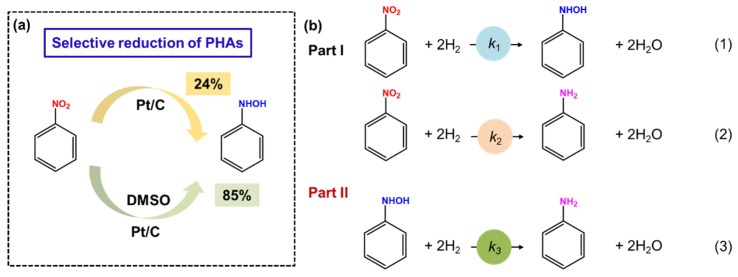
(**a**) Selective reduction of PHAs under the action of DMSO. (**b**) Several pathways in the hydrogenation of nitrobenzene. Reproduced with permission from Ref. [64]. Copyright 1987 American Chemical Society.

Catalytic hydrogenation using H_2_ as H resources dates back to 1970, when Rylander and coworkers found that carbon-supported Pt could catalyze the hydrogenation reaction of nitrobenzene to prepare N-PHA in the presence of DMSO [22]. Various influencing factors on N-PHA production have been investigated, including metal loading, metal concentration, and different carriers. The research revealed that Pt species, a carbon-based matrix, and lower metal concentrations are crucial for producing N-PHA. Note that DMSO is essential for such a reaction process. The yield of N-PHA can be up to 85%; however, only a selectivity of 24% for N-PHA was obtained in the absence of DMSO (Figure 3a).

The iridium catalyst was prepared using a modified Adams method, which hydrogenates nitro compounds to PHAs in an 80% ethanol aqueous solution [63]. Interestingly, Pt- or Pd-based catalysts prepared using the same Adams method can only yield aniline as the product. By contrast, in the hydrogenation of nitro compounds upon the iridium catalyst, the reaction preferred to consume 2 moles of hydrogen to produce PHAs at the initial stage. After that, the reaction rate decreased remarkably, exhibiting a high selectivity of PHAs. Unfortunately, the catalytic activity is poor. Subsequently, Karwa and coworkers investigated the hydrogenation of nitrobenzene to N-PHA using Pt/C as a catalyst [64]. It was found that N-PHA and aniline were generated simultaneously during the hydrogenation process. Compared with N-PHA, nitrobenzene exhibited stronger adsorption onto Pt/C, which significantly inhibited the transformation of N-PHA into aniline until it was completely depleted. As outlined in Figure 3b, DMSO could controllably suppress Reaction 2 in Part I. Meanwhile, compared with N-PHA, the nitrobenzene can preferentially adsorb on the catalysts, and, thus, Reaction 3 is completely inhibited. As a result, N-PHA was the main product. Therefore, the key to improving the selectivity of N-PHA is to prevent the direct conversion of nitrobenzene to aniline. Moreover, the effects of the solvent dielectric constant and that of substituent groups on the benzene ring were further examined. The result shows that solvents of high dielectric constants (e.g., methanol) and the nitro compounds with electron-withdrawing substituent groups favor PHAs’ production. Unfortunately, despite the improvement in selectivity, the addition of DMSO hindered the catalytic activity and restricted the application.

In addition, Takenaka and coworkers explored amines as an assisting reagent to alleviate the negative impact of dimethyl sulfoxide on catalytic activity. Under the combined action of DMSO and amines, the hydrogenation selectivity of N-PHA was up to 99% [65]. Specifically, the hydrogenation reduction was carried out on Pt/SiO_2_, affording N-PHA in a 99% yield. Subsequent research indicates that the promotive effect of amines is due to the activation of the nitro group by the amine proton, partly assisted by the increased hydride character of hydrogen on platinum. This enhancement ultimately leads to increased catalytic activity. Meanwhile, DMSO blocks the adsorption sites on the platinum surface that are easily accessible to N-PHA, preventing the continuous hydrogenation of N-PHA. The combined effect of DMSO and amine promotes high selectivity and activity in the hydrogenation of nitrobenzene. Then, the impact of the amine structure on the hydrogenation activity and selectivity was explored, and the researchers found that the promotion effect of different types of amines is as follows: tertiary amine < secondary amine < primary amine. Subsequently, a supported palladium catalyst was used to selectively hydrogenate nitro compounds into PHA, and it was found that temperature is a key factor affecting PHAs’ selectivity [66]. The catalytic systems work well in different solvents and can be applied to nitro substrates containing various functional groups. It is also found that the support structure of the catalyst also affects the hydrogenation of nitro compounds to PHAs [67].

**Figure 4:**
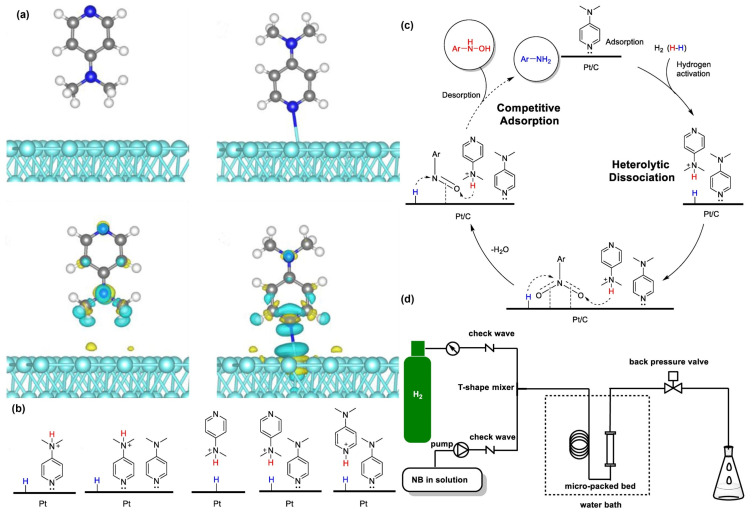
(**a**) Structures and charge difference of adsorbed DMAP models, and (**b**) the hypothetical formation mechanism of FLP, (**c**) the mechanism for Pt-catalyzed catalytic hydrogenation of nitroarene into N-PHA, and (**d**) continuous-flow reactor setup of hydrogenation of nitroarenes. Reproduced with permission from Ref. [68]. Copyright 2023 Multidisciplinary Digital Publishing Institute.

Particularly, Chen et al. found that dimethylaminopyridine (DMAP) can serve as an additive that boosts the conversion of nitrobenzene and enhances the selectivity of N-PHA without DMSO [68]. The adsorption energy of DMAP on the catalyst is similar to that of N-PHA. Thus, DMAP can cover the catalyst surface early in the reaction, making it difficult for N-PHA to reach the active center during competitive adsorption. On the other hand, DMAP can facilitate the heterolytic splitting of H_2_ on Pt/C. The nitrogen components of DMAP can serve as alkaline ligands that interact with the active site of Pt/C, forming a hindered Lewis pair (FLP) (Figure 4a,c). The heightened alkalinity plays a favorable role in enhancing the heterologous cleavage of H_2_. The dual role of DMAP could help improve the catalytic efficiency and specificity. In addition, the work achieved the preparation of PHAs using continuous-flow technology (Figure 4d). Moreover, the work has practical significance in the hydrogenation of nitro compounds; it can reduce nitro compounds containing various useful functional groups, including electron-donating groups, carbonyls, and halogens, into PHAs.

**Figure 5:**
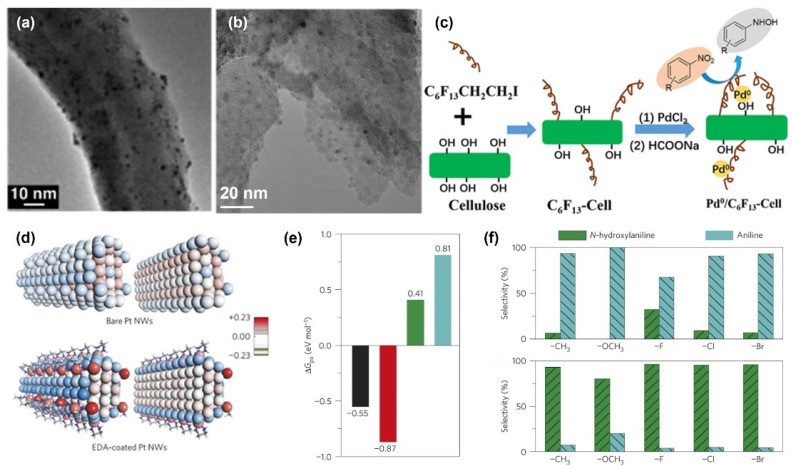
(**a**) TEM image of Pt/N-CNF-H, reproduced with permission from Ref. [72]. Copyright 2011 Wiley-VCH. (**b**) TEM image and (**c**) synthetic methods of modified cellulose carrier-loaded Pd-catalyst, reproduced with permission from Ref. [73]. Copyright 2020 Elsevier. (**d**) The surface electronic effect induced by EDA and (**e**,**f**) mechanism of catalytic selectivity of EDA-Pt NWs, reproduced with permission from Ref. [74]. Copyright 2016 Springer Nature.

Note that the aforementioned work mainly focused on the effects of exogenous additives and the mechanisms of hydrogenation of nitrobenzene to produce PHAs. Additives typically function by competing with PHAs for adsorption, thereby occupying the catalytic active sites and enhancing selectivity. Such additives can be regarded as the surface modification of the catalyst [69]. Besides additives, other surface-modification strategies, such as heteroatom modification (introducing N, B, P, etc.) and organic ligand modification [70], have also been employed to improve the catalytic selectivity towards PHAs. Introducing heteroatoms to prepare poisoned catalysts can also enhance the selectivity towards PHAs at the expense of some activity [71]. Motoyama et al. illustrated a concept by thermally decomposing platinum nanoparticles supported on nitrogen-doped carbon nanofibers (N-CNF-H) in the presence of N-CNF-H [Pt(dba)_2_] (dba: dibenzylideneacetone) to synthesize reusable poisoning catalysts (Figure 5a) [72]. The gained catalysts can selectively hydrogenate nitro compounds containing various functionalized groups to obtain corresponding aniline and PHAs. The experimental results indicate that S, N, and P can poison the catalyst, thereby reducing the reaction rate and hence improving the hydrogenation selectivity of PHAs. Furthermore, Li et al. grafted 1H,1H,2H,2H-perfluorohexyl iodide onto cellulose and introduced fluorine-containing groups to alter the surface properties of the cellulose (hydrophilicity/hydrophobicity) and then loaded Pd for the selective hydrogenation of nitrobenzene with good selectivity for N-PHA (Figure 5b,c) [73]. By studying the adsorption curves of nitrobenzene and N-PHA on the catalyst, the results show that surface properties were crucial for the selective reduction of nitrobenzene. The hydrophobic surface of the catalyst would enrich the hydrophobic nitrobenzene and repulse the adsorption of the hydrophilic N-PHA, which prevented the further reduction of N-PHA. Therefore, a high yield of N-PHA could be obtained. However, achieving excellent selectivity for PHAs containing electron-donating groups was still challenging. Chen and coworkers employed ethylenediamine as an organic surface modifier to manipulate the properties of Pt nanowire catalysts [74]. The modification facilitated the transfer of electrons to the Pt nanowires, making their surface highly electron-rich (Figure 5d). Data analysis and density functional theory (DFT) calculations demonstrated that this alteration in the interfacial electronic structure enhances the adsorption of electron-deficient reactants while preventing the over-hydrogenation of the electron-rich product PHAs (Figure 5e,f). As a result, the selectivity for PHAs significantly increased, reaching nearly 100% within 50 min in the absence of extra additives. Moreover, the catalyst maintained its selectivity without causing PHAs over-hydrogenation even after prolonging the reaction time to 2 h. The research highlighted the pivotal role of the electronic effect in determining PHAs selectivity, emphasizing the importance of modifying the electronic structure of metal nano-catalysts to boost their catalytic performance.

The bimetallic strategy is also an effective strategy to improve the catalytic performance, as the interaction between the two metals affects the electronic density and charge distribution of active centers [75]. Galvagno and coworkers developed bimetallic catalysts containing Pt-Sn by impregnating nylon powder with specific concentrations of chloroplatinic acid and tin chloride solution [76]. The catalyst exhibited a 65% selectivity towards N-aryl hydroxylamine at 80% nitrobenzene conversion, using hydrogen as the hydrogen source. Notably, the reaction temperature must be maintained at 0 °C, and the efficiency diminishes significantly as the Pt/Sn ratio is elevated. Similarly, Neri et al. prepared a carbon-supported bimetallic catalyst for the selective hydrogenation of 2,4-dinitrotoluene, where a secondary element other than Pd (e.g., Fe, Sn, or Ca) was utilized as a promoter, either by coating the palladium particles, covering them, or forming an alloy with Pd [77]. It was found that the introduced Ca could enhance the selectivity of the hydrogenation process, while Fe had the opposite effect. In addition, the size of the Pd particle also played a crucial role in influencing the catalyst’s activity and selectivity. Ca-doped catalysts have larger metal particles, which improve the selectivity for 2-hydroxy-4-nitrotoluene and its isomers, achieving a selectivity of 88% for the corresponding PHAs at a conversion rate of 50%.

Nitro compounds containing electron-withdrawing groups are more advantageous than those containing electron-donating groups in affording PHAs. However, the mechanism was still unclear. Rong et al. conducted a tentative exploration using carbon-loaded platinum colloids as the catalyst and m-dinitrobenzene as a model substrate [78]. It was found that when there is an electron-withdrawing substituent on the benzene ring of nitroaromatics (NA), the electron-withdrawing group enhances the N-O bond on the hydroxylamino group through the π-conjugation of the phenyl ring, which effectively stabilizes the PHAs and retards the further conversion of the PHAs. The influences of solvents were also studied. Intriguingly, tetrahydrofuran is much preferred in generating PHAs than ethanol and methanol, contrary to previous reports that ethanol or methanol possesses a larger dielectric constant and favors the conversion of nitro compounds to PHAs.

The catalytic system using the noble metal as the catalysts and H_2_ as reducing agent received wide attention in catalytic reduction of nitrobenzene to prepare PHAs due to its activity and selectivity. However, it still has challenges in achieving its industrial applications, possibly for the following reasons. Noble metal-based catalysts are of high activity, making them prone to excessive hydrogenation to produce aniline. Therefore, controlling the ratio of reductants and substrates makes it difficult to achieve high selectivity for PHAs. Correspondingly, the precise regulation of reaction parameters, such as temperature, pressure, and duration, is essential to enhance the selectivity but is tedious, restricting their industrial applications. Moreover, noble metals suffer limited accessibility and affordability and are susceptible to poisoning caused by nitrogen-containing solvents or other impurities, leading to catalyst deactivation.

Overall, in contrast to the extensive investigations on the hydrogenation of nitro compounds for aniline production, the synthesis of PHAs by catalytic hydrogenation using H_2_ as the H donor presents numerous challenges related to catalyst preparation, hydrogenation efficiency, and mechanistic elucidation, necessitating additional comprehensive exploration.

#### 5.1.2. N_2_H_4_ Reducing Agent

The storage and transportation of hydrazine hydrate do not require high pressure or low temperature, and its safety is higher than that of hydrogen. Hydrazine hydrate, as a hydrogen donor, is widely used to reduce nitroaromatics. Extensive research has been conducted on using hydrazine as a reducing agent for catalysts based on noble metals. The decomposition of hydrazine hydrate goes smoothly under normal temperature and pressure conditions, with the reaction rate and hydrogen release quantity being adjustable by manipulating factors such as the stoichiometric ratio between the hydrazine hydrate and the substrates. Additionally, the reaction activity associated with hydrazine hydrate is relatively high.

**Figure 6:**
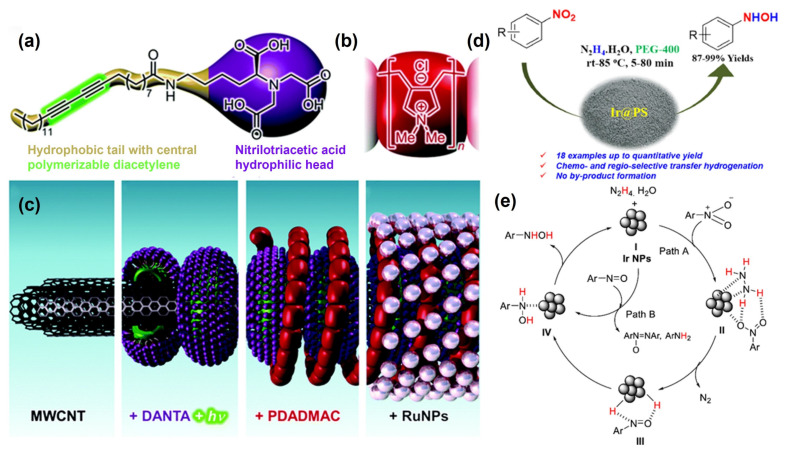
Structures of (**a**) DANTA and (**b**) PDADMAC, and (**c**) Layer-by-layer assembly of the RuCNT, reproduced with permission from Ref. [80]. Copyright 2015 The Royal Society of Chemistry. (**d**) Catalytic process and (**e**) proposed mechanism of semi-hydrogenation of nitroarenes catalyzed by Ir@PS, reproduced with permission from Ref. [84]. Copyright 2020 Britannica.

Developing a chemical-selective method of reducing nitroarenes to PHAs without additives is a common demand in industry. Shil et al. found that hydrazine hydrate could work as an H resource for solid-loaded platinum (0) (SS-Pt) nanoparticles catalyzed nitroaromatics reduction to prepare PHAs [79]. Under optimal conditions, the yield of PHAs achieved 92%. This system has broad applicability for selectively reducing various nitroaromatics with different reducible functional groups to the corresponding hydroxylamines, with corresponding PHA yields ranging from 75% to 98%. After that, Doris et al. reported a carbon nanotube–ruthenium nanohybrid catalyst that could achieve the selective conversion of 4-bromo-nitrobenzene to corresponding PHAs by using hydrazine monohydrate as the hydrogen source [80]. Diacetylene nitrilotriacetic amphiphile (DANTA) and poly diallyl dimethylammonium chloride (PDDMAC) layers are gradually assembled on the multi-walled carbon nanotube (MWCNT), and the Ru was able to stably anchor on the surface of the modified supports, effectively improving the dispersion of active centers (Figure 6a,c). Thus, the reaction went smoothly at room temperature, affording PHAs’ 98% yield as the sole product. After five cycles, there was only a slight decrease in activity. Further investigation shows that the selectivity of PHAs was highly dependent on the solvent.

When metal nanoparticles are loaded onto the organic polymers, the nature of the polymer carrier affects the dispersion of nanoparticles in organic solvents [81], which can be an effective strategy for controlling the selectivity of PHAs. Tyler et al. prepared soluble polystyrene-loaded Ru nanoparticles capable of reducing nitroaromatic hydrocarbons to PHAs in high yield and selectivity under the mediation of hydrazine [82]. An isolated yield of PHAs of 88% was observed within 1 h at room temperature. The selectivity of the catalyst towards PHAs was highly related to the polarity of the solvent and the solubility of the nanoparticle catalyst. The solvent-dependent alteration in selectivity provides a high degree of flexibility in the catalytic process.

Similarly, the chemoselective hydrogenation of nitroaromatics can be carried out over poly(N-vinyl-2-pyrrolidone) (PVP)-stabilized iridium nanoparticles (PVP[Ir]) [83]. Colloidal iridium nanoparticles exhibited good functional group tolerance in the hydrogenation of nitroaromatics to anilines [84]. Hydrazine hydrate was chosen as the reduction source, and PEG-400 as the green solvent, together affording a good yield of PHAs (Figure 6d,e). The catalysts show excellent reactivity, broad substrate applicability, and compatibility with a range of reducible functional groups.

Notably, unlike using hydrogen as a reducing agent, hydrazine hydrate reducing agents do not require additional additives, such as DMSO, or amines. Exploring the underlying mechanisms is of great significance for developing and designing subsequent catalytic systems. Meanwhile, the reduction system still requires the precise adjustment of reaction parameters to improve selectivity, and related research is not in-depth enough, requiring more comprehensive exploration in mechanism elucidation.

#### 5.1.3. Borohydride Reducing Agent

Borohydride is a common hydrogen donor in catalytic transfer hydrogenation. Compared to H_2_ and hydrazine hydrate, borohydride has higher stability and hydrogen content and is an outstanding hydrogen-storage material.

**Figure 7:**
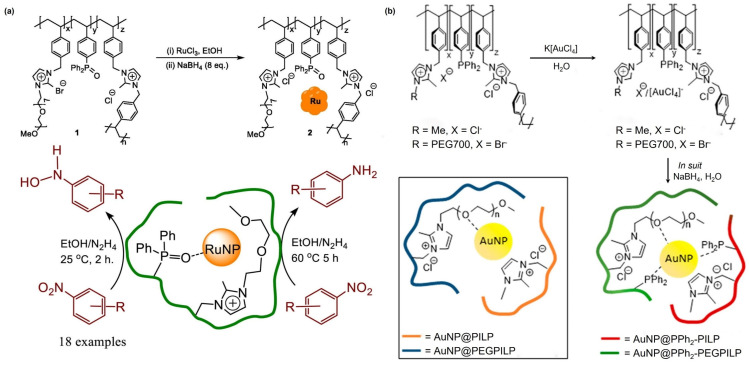
(**a**) Synthesis and composition of PIIL-stabilized ruthenium nanoparticles, reproduced with permission from Ref. [85]. Copyright 2022 Elsevier. (**b**) Composition of polymers and schematic diagram of PEG and PIILP-stabilized gold nanoparticles, reproduced with permission from Ref. [86]. Copyright 2019 American Chemical Society.

Paterson et al. prepared a polymer-immobilized ionic liquid-stabilized nano-Ru catalyst [85]. First, the polymer was decorated by phosphine oxide, and Ru was loaded onto the modified polymers by the impregnation method and then reduced in situ with an excess of NaBH_4_ to afford the catalyst (Figure 7a). The catalyst can efficiently and selectively reduce various N-aryl and N-heteroaryl nitroaromatic hydrocarbons to corresponding PHAs in ethanol, with the selectivity of PHAs exceeding 99%. Nevertheless, the catalyst is unsuitable for nitro-compounds with electron-rich substituents, resulting in a slow reduction process, with aniline as the main product.

Similarly, Doherty and coworkers developed phosphorus-modified polymer-immobilized ionic liquid-stabilized gold nanoparticles for the catalytic reduction of nitrobenzene to prepare N-PHA using sodium borohydride (Figure 7b) [86]. The reaction was conducted in water under a nitrogen atmosphere, resulting in a 96% yield of N-PHA at full conversion of nitrobenzene within 6 h. By contrast, azobenzene was produced as the sole product when ethanol was employed as the solvent, and the catalytic system can selectively reduce nitroaromatics to important intermediates (azobenzene, hydroxylamine, and aniline) by varying the conditions during the reaction process. The results indicate that the selectivity of N-PHA decreases with the increasing reaction temperature. This temperature-dependent selectivity trend suggests that the activation barrier of the catalyst for reducing N-PHA to aniline may be higher than that for reducing nitrobenzene to N-PHA. Consequently, achieving higher selectivity for N-PHA at lower temperatures is more feasible. It also found that the introduction of polyethylene glycol (PEG) and diphenylphosphine onto the catalyst resulted in a dramatic improvement in the conversion rate and selectivity of N-PHA. The results can be attributed to the hydrophilicity of PEG to improve the water solubility and/or the dispersion of the catalysts and the addition of diphenylphosphine to significantly increase the activity of the reaction, thus improving the efficiency.

**Figure 8:**
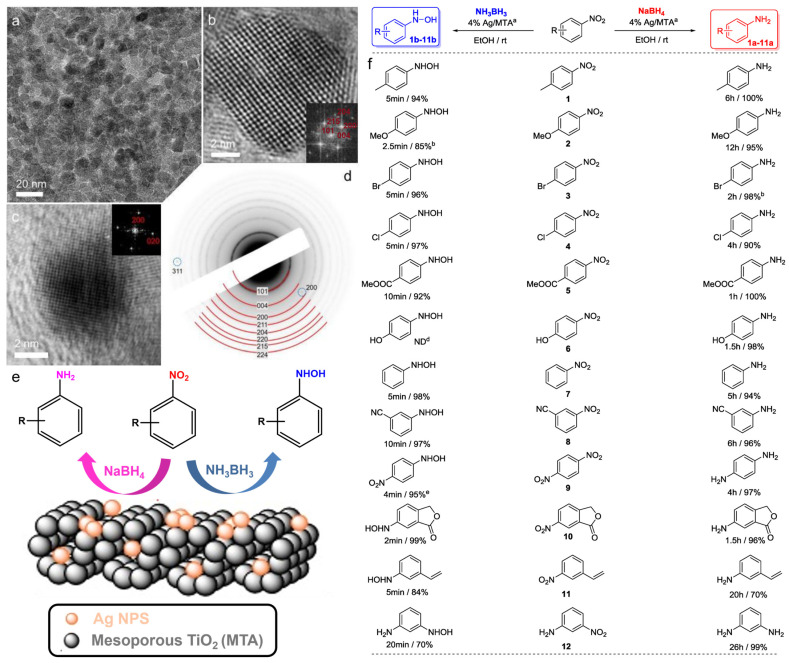
(**a**) TEM image. HRTEM of (**b**) TiO_2_ and (**c**) Ag nanoparticle. (**d**) SAED pattern of mesoporous 4% Ag/MTA catalyst, the red curve can be attributed to the rutile phase of TiO_2_, while the blue mark can be labelled as the (200) and (311) reflections of cubic Ag. (**e**) Selective reduction of Ag/MTA catalyst to aniline and PHAs. (**f**) Chemoselective reduction of nitroarenes into amines and PHAs. Reproduced with permission from Ref. [87]. Copyright 2016 Multidisciplinary Digital Publishing Institute.

Ag-based catalysts have received much attention due to their superior catalytic activity compared with other noble metal-based catalysts. For example, Andreou and coworkers prepared Ag/MTA composite materials via the photochemical deposition of AgNPs on mesoporous titanium dioxide (MTA) surface (Figure 8a,d) [87]. Ag/MTA can catalyze the reduction of nitro compounds to the corresponding anilines and PHA under relatively mild reaction conditions with sodium borohydride and NH_3_BH_3_ as the reductants, respectively. A series of nitroaromatics were examined to investigate the substrate suitability of this reduction process. It can be observed that PHAs can be obtained in high yields (>84%), in a short period (2–10 min), with NH_3_BH_3_ as the reductant (Figure 8e), suggesting that the Ag/MTA-NH_3_BH_3_ catalyst can be applied to a wide range of aromatic nitro compound for preparing the corresponding PHAs (Figure 8f). In addition, the 4% Ag/MTA catalysts deliver decent reusability, and they could be used at least three times without significant loss of catalytic activity and selectivity.

In the catalytic system using noble metals as catalysts and borohydride as hydrogen sources, mild reaction conditions and relatively good catalytic performance are exhibited. Like the catalytic system using hydrazine hydrate as the hydrogen source, it does not require the introduction of external additives, but it still requires strict control of reaction conditions to achieve higher selectivity and has a limited substrate scope. The research on related mechanisms is still relatively shallow, and developing excellent catalysts and exploring their hydrogenation mechanisms is a worthwhile topic.

### 5.2. Non-Noble-Metal Catalysts

Noble-metal catalysts have great advantages in activity, but they are expensive and resource scarce, and they require strict control of reaction conditions to obtain highly selective PHAs. Although non-noble-metal catalysts usually possess lower activity and selectivity, they are recently attracting increasing attention due to their low cost and abundant resources.

#### 5.2.1. H_2_ Reducing Agent

Nickel is recognized for its effective hydrogen-dissociation capabilities. Particularly, Raney nickel catalysts have been widely utilized in catalytic hydrogenation [88]. However, Raney nickel itself exhibits limited selectivity. As a result, diverse strategies were proposed to improve the catalytic performance of Raney nickel for producing PHAs. Xu and coworkers developed passivation-treated Raney nickel by ammonia/DMSO (1:10, *v*/*v*) [89]. This, together with continuous flow reaction technology, affords a remarkable conversion rate (99.4%) and selectivity (99.8%) of PHAs. However, the efficiency and selectivity of PHAs production in the continuous flow system are notably influenced by the reaction duration. Shorter reaction times result in lower conversion rates but higher selectivity, while longer reaction times lead to increased conversion rates but reduced selectivity. Furthermore, the impact of catalyst particle size was investigated, revealing that catalysts with larger surface areas exhibited a better performance due to enhanced passivation effects. A proposed reaction mechanism for this continuous flow system involves the adsorption of aromatic nitro compounds on the metal catalyst surface, coordination of the nitrogen–oxygen double bonds (within nitro groups) with the metal, subsequent hydrogen dissociation, and direct hydrogenation of active hydrogen into the nitrogen–oxygen bonds to form hydroxylamine compounds. An evaluation of substrate compatibility demonstrated that compounds containing halogen or benzyl groups yielded PHAs with satisfactory yields. In contrast, the ester groups significantly inhibited the conversion and selectivity for hydroxylamine production.

The introduction of a second metal was also found to be an effective strategy for improving the catalytic performance of Raney nickel [90]. For instance, Studer et al. reported that Raney nickel affords a hydroxylamine selectivity of 70% [91]; further investigations demonstrated that vanadium, as a promoter, was able to modulate hydroxylamine stacking, and the N-PHA selectivity could be increased to 86%. Unfortunately, among catalytic systems with a non-noble metal as the catalyst and H_2_ as the reducing agent, only nickel-based catalysts were shown to effectively hydrogenate nitro compounds to PHAs, while other non-noble metals remain unexplored. This is possibly due to the low activity of non-noble metals, which demand high temperatures and pressure for hydrogenation. The energy barriers for the hydrogenation of nitrobenzene to N-PHA and aniline are −1.57 eV and −2.56 eV [92], respectively. Thus, it is thermodynamically more favorable to afford anilines, whereas high temperature and pressure hinder the synthesis of PHAs. Therefore, developing non-noble-metal catalysts in the hydrogen system is a great challenge.

#### 5.2.2. N_2_H_4_ Reducing Agent

Ayyangar and coworkers utilized Raney nickel as a catalyst and hydrazine hydrate as a reducing agent to facilitate the reduction of nitro-aromatic hydrocarbons to PHAs [93]. This process can reduce most nitro compounds to corresponding PHAs in a solvent mixture containing 1:1 ethanol and dichloroethane and partially reduce dinitroaromatic hydrocarbons to nitroaniline. The specificity of this method was contingent upon the temperature, necessitating maintenance of the reaction temperature within the range from 0 to 10 °C; a lower temperature (below 0 °C) does not support the catalytic reaction. Besides Ni, Mg-based catalysts were also studied. Pasha et al. achieved the selective reduction of various substituted nitroaromatic hydrocarbons using Mg as a catalyst and hydrazine sulfate as a reducing agent [94]. The reaction was performed in water, affording an N-PHA yield of over 90% within a reduction time of 1.5 min. The product separation did not require organic solvents, making this method fast, environmentally friendly, simple, and effective.

The advantage of hydrazine is that it can be used as both a hydrogen source and a nitrogen source. The non-noble-metal–hydrazine-hydrate system is environmentally friendly, easy to operate, and can achieve high activity and selectivity. In the synthesis of PHAs, exogenous additives are often needed, generally N-containing compounds; meanwhile, hydrazine hydrate-involved catalytic systems usually achieve higher PHA yields without the assistance of additives.

#### 5.2.3. Borohydride Reducing Agent

Ren et al. prepared N-PHA by hydrogenating nitrobenzene with antimony powder as the catalysts and sodium borohydride as the reductant, achieving a high yield of N-PHA (88%), without over-reduction product [95]. The KBH_4_/BiCl_3_ system has also been found to reduce nitroaromatics to the corresponding PHAs with high yield and selectivity, especially for those with electron-withdrawing groups [96]. Moreover, the mechanism lying behind the difference in reducibility for nitro compounds containing different substituted groups was elucidated. Electron-donating substituents impeded the reduction of aromatic nitro compounds, while electron-withdrawing substituents promoted the selective reduction of nitro compounds to corresponding PHAs.

The non-noble-metal borohydride catalysts exhibit good hydrogenation selectivity and activity but are limited by the type of substrate and cannot effectively hydrogenate nitro compounds with electron-donating groups. Therefore, developing catalytic systems with a broader range of substrates is a future research direction.

### 5.3. Non-Metallic Catalysts

#### 5.3.1. N_2_H_4_ Reducing Agent

**Figure 9:**
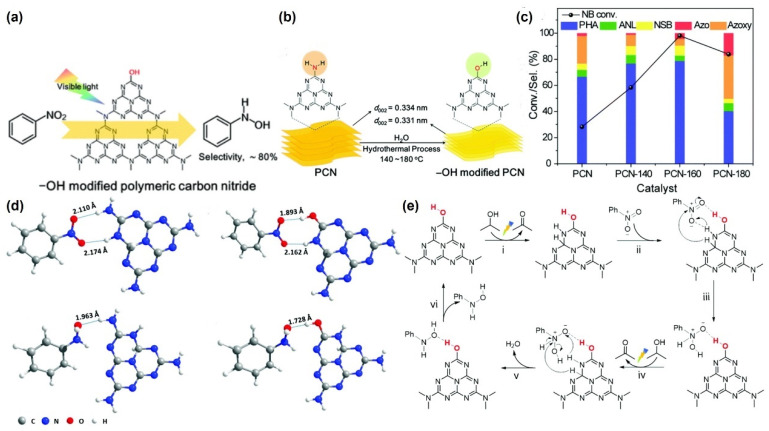
(**a**,**b**) Schematic representation for the formation of -OH-modified PCN under hydrothermal conditions. (**c**) Photocatalytic activity for the selective reduction of nitrobenzene. (**d**) Analysis of the reaction mechanism. (**e**) Mechanism for the hydrogenation of nitrobenzene over the PCN-160 surface. Reproduced with permission from Ref. [97]. Copyright 2021 The Royal Society of Chemistry.

Pei et al. developed polycarbonate nitride possessing enriched -OH groups as catalysts for nitro compounds for preparing PHAs (Figure 9a,b) [97]. Using isopropyl alcohol/water as a solvent and visible-light irradiation, the catalysts delivered an excellent reaction rate and achieved 80% PHA selectivity (Figure 9c). The -OH groups can enhance the adsorption of nitrobenzene and reduce the photoelectron–hole pair recombination, thus facilitating the photocatalytic oxidation of isopropyl alcohol and providing more protons for hydrogen transfer (Figure 9d,e). Moreover, the modification of the -OH groups raised the conduction band position and supplied high-energy photogenerated electrons to reduce nitrobenzene. The system favored the hydrogenation of nitro compounds with electron-withdrawing groups, while it was hindered by electron-donating groups.

In non-metallic-N_2_H_4_ catalytic systems, non-metallic catalytic systems are commonly catalytically inert and, thus, have to resort to strong reducing agents and extra force (e.g., light and electricity) to finish the hydrogenation process, and they are always applicable to a limited range of substrates. The current related research is very limited, but based on the environmentally friendly nature of non-metallic materials, it is of great significance to develop efficient non-metallic catalytic systems.

#### 5.3.2. Borohydride Reducing Agent

Yanada et al. reported that aromatic nitro compounds could be reduced to their corresponding PHAs in ethanol solution at room temperature using selenium powder as a catalyst and sodium borohydride as a reductant [98]. However, the same product was not obtained when water was used as a solvent instead of ethanol. The active species for the selenium-catalyzed reduction is the hydrogen selenide anion. The reduction of nitro compounds with different substituents has also been examined; the results showed that the reaction was faster for substrates with electron-withdrawing substituents and completely inert for those with electron-donating groups.

Currently, non-metallic catalytic systems demonstrate restricted efficacy, with most research efforts confined to laboratory settings and distant from practical industrial implementation. Moreover, its efficacy is confined to a restricted range of substrates, and nitro compounds containing electron-donating groups exhibit low conversion rates to PHAs. However, the non-metallic catalytic system is user-friendly and cost-effective. Therefore, the research into and development of an effective non-metallic catalyst for the selective hydrogenation of nitroaromatic compounds hold considerable importance in the development of green chemistry.

### 5.4. Biological Catalysis

The biosynthesis of PHA compounds from nitroaromatic hydrocarbons involves the metabolic conversion of nitrobenzene compounds into the desired products using plant cells, baker’s yeast [99], and biocatalytic enzymes. In 2004, Li et al. were the first to prepare PHAs via a bioprocess using baker’s yeast as the catalyst and p-dinitrobenzene as the model substrate [100], achieving a 100% conversion rate and a corresponding PHA yield of 95%. However, this system has substrate limitations: the reaction rate and selectivity of nitroaromatics with electron-withdrawing groups were significantly improved, but the nitroaromatics with electron-donating groups (methyl and methoxy) were not reduced.

**Figure 10:**
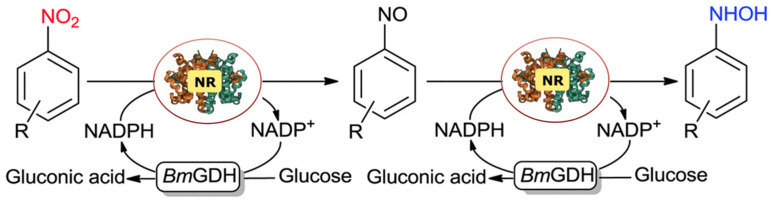
Proposed bioreaction pathways for the controllable reduction of nitroarene compounds to the corresponding PHAs. Reproduced with permission from Ref. [102]. Copyright 2014 The Royal Society of Chemistry.

Nitroreductases (NTRs), found in bacteria and a few eukaryotes, are flavin-containing enzymes that utilize NADH or NADPH as an electron source to reduce nitroaromatics to the corresponding PHAs and amines [101]. They are considered ideal biocatalysts for the synthesis of PHAs and amines. Xu et al. utilized a novel nitroreductase, BaNTR1, to catalytically reduce nitroaromatic hydrocarbons for the synthesis of PHAs (Figure 10) [102]. The reaction achieved 100% conversion and >99% PHA selectivity with 1 h of reaction and at a pH of 7. Unlike most current reactions that require strict control of the reaction time or the addition of toxicants to obtain pure PHAs, this catalytic system still maintained >99% PHA selectivity even when in operation for 24 h. Moreover, this system could efficiently and selectively hydrogenate nitroaromatics with electron-withdrawing groups to PHAs, and the nitroaromatics with electron-donating groups were poorly reactive and produced various by-products, a phenomenon also observed in other studies. The PHAs from nitroaromatics with electron-withdrawing groups were relatively stable [16], while the PHAs from nitroaromatics without such groups were unstable and quickly formed azobenzene.

Li et al. used various sources of plant cells for nitro compound reduction [100]. The grape plant cells had a very high selectivity for N-PHA. The reaction-time effect was different from typical enzyme-catalyzed reactions. It barely occurred on the first day, but the conversion rate rose to 73% after 2 days. After 4 days of reaction, the conversion rate reached 96%, and the selectivity remained above 98%. This biological reduction method was simple, convenient, and efficient, but it had a narrow scope and was only effective for a few nitro compounds.

The biocatalyst used in the bioreduction method is non-polluting and conveniently available, but the formulation cost is high, the separation and purification of the resulting product is difficult, and the reproducibility of the biological cells is poor, thus making it difficult to produce on a large scale. Furthermore, Table 2 summarizes the main investigation results related to the preparation of PHAs by catalytic reduction of nitro compounds. Through these examples, the differences in catalysts, reaction conditions, and catalytic performance in various systems can be more intuitively presented. The noble-metal–H_2_ catalytic system is currently the most widely studied hydrogenation system, with mild reaction conditions and good catalytic performance. However, it usually requires the introduction of external additives, and the catalytic system has obvious solvent dependence. Non-noble-metal catalysts, non-metallic catalysts, and biocatalytic systems have the advantage of relatively low cost, but they usually exhibit relatively harsh reaction conditions (a longer reaction time and higher temperature).

## 6. Investigation of the Reduction Mechanism

**Scheme 4:**
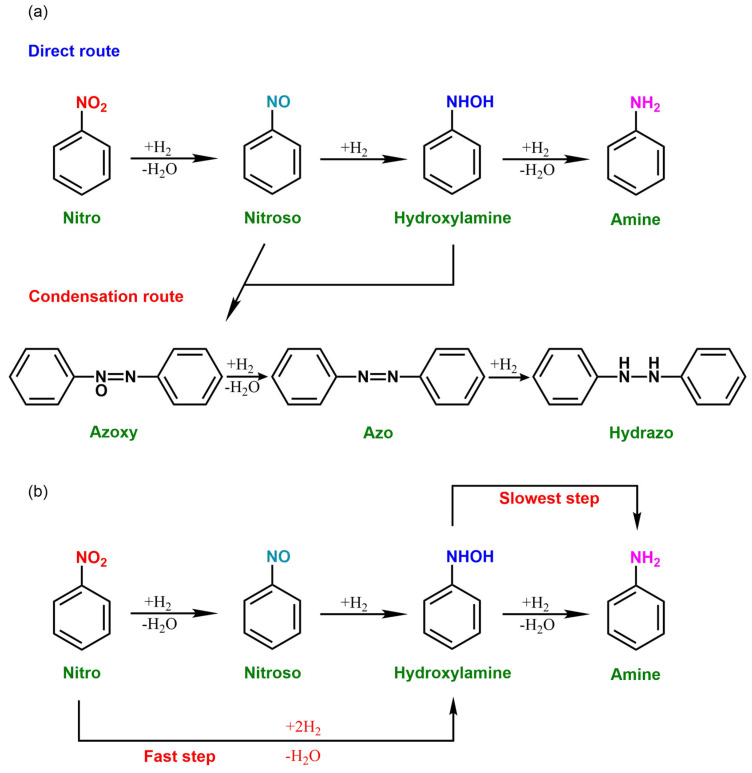
(**a**) The reaction pathways for the reduction of an aromatic nitro compound to the corresponding aniline. (**b**) The reaction pathway for the preparation of aniline derivatives by hydrogenation of nitrobenzene over Au/TiO_2_ catalyst.

The catalytic hydrogenation of nitrobenzenes is a common sequential process. The established reaction pathway for the catalytic reduction of aromatic nitro compounds is based on the model introduced by Haber and Takenaka (Scheme 4a). This reaction mechanism proposes two distinct pathways. In the primary pathway, aromatic nitro compounds are first reduced to nitroso compounds, which are subsequently reduced to PHAs [103,104]. The PHAs then undergo extensive hydrogenation to produce aniline derivatives. In the secondary pathway, nitroso compounds react with PHAs to generate an azo compound, which undergoes a reduction in a series of successive steps to form an azobenzene, a hydrogenated azobenzene, and ultimately an aniline.

Afterwards, the Haber mechanism was questioned, and it was controversial whether nitrosobenzene was a necessary intermediate for the hydrogenation of nitrobenzene to produce N-PHA and aniline. Jackson observed variations in the reaction profiles when nitrosobenzene was utilized as the initial substrate, indicating that nitrosobenzene may not be an essential intermediate. This suggests the existence of a potential direct pathway from nitro compounds to hydroxylamine. Corma and coworkers further ascertained the conclusion through experiments, proposing that aromatic nitro compounds undergo hydrogenation following the reaction pathway outlined in Scheme 4b. Moreover, hydroxylamines tend to undergo self-disproportionation upon exposure to heat, posing challenges in achieving high yields of PHAs.

The process of the catalytic hydrogenation of nitrobenzenes for the synthesis of PHAs involves multiple stages (Scheme 4) [20]. The determining step is the adsorption rate of hydrogen and nitrobenzene on the catalyst surface [105]. The series of reaction stages comprises the transport of nitrobenzene and hydrogen molecules to the solid catalyst surface, their absorption onto the active sites of the catalyst surface through physical or chemical means, the initiation of a chemical reaction between the absorbed reactant molecules on the catalyst surface, and the subsequent release of product molecules from the catalyst surface, succeeded by their dispersion away through diffusion. During this process, nitrobenzene and nitrosobenzene can simultaneously form N-PHA. The gradual accumulation of N-PHA on the catalyst surface indicates that converting N-PHA to aniline is a decisive step in the hydrogenation of nitrobenzene to aniline. Therefore, inhibiting this step is an important prerequisite for controlling the high yield of PHAs. Studies have shown that regulating the electronic state of catalysts can affect the adsorption of N-PHA, thereby blocking its continued hydrogenation and enhancing the selectivity of N-PHA. Some studies have also found that by regulating the surface density of modifiers to expose specific locations of active sites, intermediates can bind to the active sites with their long axes perpendicular to each other, enhancing N-O cleavage while making N-PHA more easily desorbed, thereby increasing the selectivity of N-PHA.

The mechanism of nitrobenzene hydrogenation to N-PHA is not yet very clear. It is necessary to establish a structure–activity relationship between the catalyst and the mechanism to guide the construction of industrial catalysts in reverse, which is essential for the development of industrial synthesis of N-PHA.

## 7. Conclusions and Perspectives

For decades, the synthesis of PHAs, widely used chemical intermediates in industry, has received continuous attention from both academia and industry. Hydroxylamine synthesis involves various methods, such as hydrolysis, oxidation, addition, and reduction. Each method has its inherent characteristics and application scope. Reducing nitro compounds represents one of the most suitable technologies for the synthesis of PHAs, and it is also the current research hotspot. The reduction process can be divided into two types: non-catalytic reduction and catalytic reduction. Non-catalytic reduction is the earliest method for preparing PHAs. It demands strict control of the stoichiometric ratio and reaction conditions. The activity and selectivity are restricted, and a large amount of salt-containing wastewater is generated, which is uneconomical and not in line with green development. Because of this, catalytic reduction has gradually become the dominant method for PHAs synthesis. In the catalytic reduction process, the selection of high-performance catalysts and suitable reducing agents is crucial. Besides H_2_, borohydrides, hydrazine compounds, alcohol compounds, and sulfides can also work as reducing agents to reduce nitro compounds into PHAs in the presence of catalysts via transfer hydrogenation. Therefore, catalytic reduction technology was classified and discussed based on different types of catalysts (including noble-metal catalysts, non-noble-metal catalysts, and non-metal catalysts) and various reducing agents.

Noble metal-based catalysts are the most extensively used for hydrogenating nitrobenzene to produce PHAs. H_2_, hydrazine hydrate, and borohydride are mainly used as reducing agents. Among them, H_2_ is the most preferred reducing agent and H donor due to its low cost and environmental friendliness. Noble metal-based catalysis systems are well established, and strict control of reaction conditions (time, pressure, and temperature) is usually required to avoid over-hydrogenation and maintain the selectivity of PHAs. Thus, how to customize catalysts to achieve catalytic systems with high selectivity and reducibility is currently a research focus, and, correspondingly, different strategies have been proposed. These strategies include the addition of toxins, alloying, ligand effects, and support regulation. However, there are still challenges in achieving industrial standards due to the poor hydrogenation performance for nitro compounds containing electron-donating groups and the dependence of catalytic systems on solvents.

Non-noble-metal catalysts for PHAs’ synthesis are mainly confined to nickel-based ones, especially Raney nickel. Other non-noble-metal catalysts or non-metallic-based catalysts usually need to perform under harsh conditions (e.g., high temperature) due to their low activity, which leads to the over-hydrogenation to produce aromatic amines. For example, Fe-, Co-, Ni-, and Cu-based catalysts have been widely used in the preparation of aniline, but the catalytic synthesis of PHA remains a challenge. Meanwhile, for non-metallic catalysts, external forces such as light, electricity, and ultrasound are crucial for promoting effective conversion processes. Unfortunately, research on this catalytic system is not yet sufficient. Finally, research on the hydrogenation synthesis of PHA from aromatic nitro compounds is currently not in-depth enough.

Based on the above discussion, the following prospects for the hydrogenation synthesis of PHA deserve attention: (1) Each catalytic system has its drawbacks and advantages, so integrating the advantages of different catalytic systems will provide a new approach for the high-efficiency catalytic hydrogenation synthesis of PHA. For example, the hydrazine hydrate-based catalytic system is easy to operate and does not require external additives, but it is expensive. The catalytic system using H_2_ as a reducing agent has high efficiency, environmental friendliness, and low cost; however, it usually requires external additives (e.g., DMSO) or operates under an alkaline condition. A suitable alkaline environment is necessary to improve the catalytic selectivity towards PHAs, and, correspondingly, designing catalysts of certain alkaline properties may be beneficial for improving the PHAs’ selectivity in H_2_-based catalytic systems. (2) The current research lacks in-depth analysis and thorough investigation of underlying mechanisms. Correspondingly, future research should be more in-depth, especially in studying the structure–activity relationship between catalysts and catalytic performance, combined with theoretical calculations and in situ technology to deeply study catalytic processes. This is essential for advancing the development of subsequent catalytic systems. (3) Most of the existing research mainly uses high-pressure reactors for hydrogenation reactions, focusing on parameter optimization during the hydrogenation process, including pressure, temperature, reactant quantity, etc., and has not progressed to the stage of pilot scale and industrial application, which is not conducive to large-scale industrial manufacturing. Future investigations should explore the implementation of continuous operations like fixed beds, align them with industrial practices, and offer theoretical underpinnings for practical production processes. (4) Issues such as inadequate stability and challenging separation methods have been identified in the production of PHAs. PHAs usually serve as intermediates for a range of high-value chemicals. Future studies should pay more attention to the direct synthesis of downstream products by using nitro compounds instead of purified PHAs as the starting materials, streamlining process steps, and implementing continuous production techniques. This approach will effectively tackle the stability and separation challenges associated with PHAs’ production and minimize manufacturing costs, in line with the strategic development of comprehensive green transformation of the economy and society.

## Figures and Tables

**Table 1:** Comparison of different synthesis systems of hydroxylamine.

No.	Method	Substrate	Catalysts	Reagents	Pressure	T (°C)	Solvent	Time	Yield (%)	Ref.
1	Alkylation	1-acetoxy-4-azidobutyl	n/a	Substitute: NH_2_OH	n/a	0	Dichloromethane	3 h	54	[23]
2	Arylation	Alkenes	n/a	Substitute: NH_2_OH	n/a	rt	Hexafluoroisopropanol	3 h	<70	[24]
3	Hydrolysis	Nitromethane	n/a	Acidifier: HCl	n/a	<120	H_2_O	4 h	90	[27]
4	Hydrolysis	Nitrone	n/a	Acidifier: oxalate	n/a	rt	MeOH	n/a	62	[28]
5	Hydrolysis	Nitrone	n/a	Acidifier: HCl	n/a	n/a	H_2_O	n/a	72	[35]
6	Oxidation	Primary amines	n/a	Oxidant: MCPBA	n/a	60	CH_3_CN	24 h	82	[28]
7	Oxidation	Primary amines	Sodium tungstate	Oxidant: UHP	n/a	rt	Et_2_O	6 h	80	[29]
8	Oxidation	Nitrosoarenes	Mes-acridinium perchlorate	External light: blue LED	n/a	rt	CH_2_Cl_2_	1 h	58	[30]
9	Reduction	Oximes	n/a	Reductant: NaBH_3_CN	n/a	rt	n/a	n/a	99	[32]
10	Reduction	Oximes	Ni(OAc)_4_·4H_2_O	Reductant: H_2_	50 bar	50	TFE/AcOH	24 h	67	[33]

**Table 2:** Comparison of different catalytic systems for catalytic reduction of nitro compounds to PHAs.

No.	Catalyst	Reducing Agent	Solvent	Additives	T (°C)	Time (min)	Sele. (PHAs, %)	Ref.
1	5%Pt-C	H_2_	Ethanol	DMSO	25	60	85.0	[22]
2	Iridium	H_2_	Ethanol	n/a	60	25	58.0	[63]
3	5%Pt-C	H_2_	Methanol	DMSO	60	45	70.0	[64]
4	5%Pt-SiO_2_	H_2_	IPA	DMSO/TEA	rt	105	98.7	[65]
5	Pd-SIO-1	H_2_	IPA	n/a	rt	120	96.0	[67]
6	5%Pt-C	H_2_	THF	DMAP	25	-	94.0	[68]
7	Pt(1%)/N-CNF-H	H_2_	Ethyl Acetate	n/a	rt	360	90.0	[72]
8	EDA-Pt NWs	H_2_	Methanol	Sodium hydroxide	25	120	>99.9	[74]
9	PdCa_3_	H_2_	Ethanol	n/a	rt	-	88.0	[77]
10	2% Pt/C	H_2_	THF	n/a	10	70	92.3	[78]
11	SS-Pt(0)	N_2_H_4_·H_2_O	PEG-400	n/a	60	190	92.0	[79]
12	RuCNT	N_2_H_4_·H_2_O	THF	n/a	rt	150	98.0	[80]
13	Ru/PS	N_2_H_4_·H_2_O	CHCl_3_	n/a	rt	45	88.0	[82]
14	Ir@PS	N_2_H_4_·H_2_O	PEG-400	n/a	85	30	87.0	[84]
15	AuNP@PEG-PIILP	NaBH_4_	Water	n/a	25	40	>99.9	[86]
16	Ag/MTA	NH_3_BH_3_	Ethanol	n/a	rt	5	94.0	[87]
17	Passivated-Reney Ni	H_2_	THF	n/a	rt	1920	93.7	[89]
18	Raney Ni	N_2_H_4_·H_2_O	C_2_H_4_Cl_2_	n/a	10	120	88.0	[93]
19	Mg	N_2_H_4_-H_2_SO_4_	Water	n/a	80	1.5	94.0	[94]
20	Sb	NaBH_4_	Methanol	n/a	rt	60	88.0	[95]
21	BiCI_3_	KBH_4_	Ethanol-H_2_O	n/a	rt	15	81.0	[96]
22	PCN-160	IPA	IPA	Visible	80	1080	80.0	[97]
23	Se	NaBH_4_	Ethanol	n/a	25	15	81.0	[98]
24	Grape-plant cells	n/a	H_2_O	n/a	25	4 Day	96.0	[100]
25	BaNTR1	n/a	H_2_O	Sodium phosphate buffer	30	60	98.7	[102]
